# 

**Published:** 1856-04

**Authors:** 


### CONTENTS.

ORIGINAL COMMUNICATIONS— PAGE.

Synopsis of Yellow Fever, with its Pathology and Treatment, as it appeared at the Marine Hospital, Quarantine, N. Y., during 1854 and 1855. By Theod. Walser, M.D., Assistant Surgeon........................ 151

Notes to serve as the basis of an Essay on the Effects of Dislocations, Sprains, and Fractures, into or near the Elbow-Joint. By J. W. Freer, M.D., Professor of Anatomy in Rush Medical College............ 161

BOOK NOTICES,..................................................................181

EDITORIAL,.......................................................................191

